# Effect of Perineal Massage on the Rate of Episiotomy 

**Published:** 2019-09

**Authors:** Farideh Akhlaghi, Zeynab Sabeti Baygi, Mohsen Miri, Mona Najaf Najafi

**Affiliations:** 1 Women's Research Center, Faculty of Medicine, Mashhad University of Medical Sciences, Mashhad, Iran; 2 Faculty of Medicine, Mashhad University of Medical Sciences, Mashhad, Iran; 3 Clinical Research Development Center, Imam Reza Hospital, Faculty of Medicine, Mashhad University of Medical Sciences, Mashhad, Iran

**Keywords:** Perineal Massage, Delivery, Episiotomy

## Abstract

**Objective:** Women frequently experience perineal damage after a vaginal delivery. This study aimed to investigate the effect of perineal massage (PM) during labor on the need for episiotomies.

**Materials and methods:** The study is a double-blind randomized clinical trial conducted with 99 patients (n=49 controls; n=50 cases). Participants comprised of nulliparous pregnant women aged from 18 to 35 years in the 37^th^-42^nd^ week of gestation, who referred to the Um-al-Banin Hospital of Mashhad from July to October 2018, for vaginal delivery and were in the active stage of labor. Allocation to study groups was based on a random allocation list generated by a software application. PM was performed for the cases in the active stage four times, each lasting for two minutes at intervals of half an hour. The massage was continued at the beginning of the second stage of labor for ten minutes. Control women received routine care. The delivery was practiced by a midwife who was blinded to the study groups and the performance or non-performance of massage. Data were analyzed in SPSS software version 16.

**Results:** The need for episiotomy was significantly lower in the PM group than in the control group (p = 0.05). Spontaneous perineal tears were significantly higher in mothers of the PM group (p = 0.05. The spontaneous tear degree in the 20 mothers who did not require episiotomy (p = 0.5) and the degree of perineal tear in mothers who needed an episiotomy (n = 79; p = 0.1) were not significantly different in the two groups. In the PM group members who did not require episiotomy (n = 14) and the mother underwent a spontaneous tear, first-degree tears were more frequent than second-degree ones. The median duration of the active stage of labor until the stage completion was lower in the PM group than in the control group, although the difference did not reach statistical significance (p = 0.3). The median of the second stage duration in the control and intervention groups were 55 and 45 minutes, respectively, where the difference was significant (p = 0.002), and the median time of completion of the active stage until delivery in the PM group had reduced.

**Conclusion:** PM had a significant impact on the reduction of the need for episiotomies and the duration of the second stage of labor. Thus, it can be suggested as a safe, simple, low-cost, and effective technique to reduce the perineal damage during delivery.

## Introduction

As a human right and a social goal, health is considered a national priority in many countries. Over 130 million infants are born worldwide every year. Labor and delivery can be associated with complications for the mother (1). Vaginal deliveries usually result in genital tract damage in primiparous women, who report complications such as short-term postpartum pain and discomfort as well as dyspareunia, than women who deliver with an intact perineum. . Therefore, various interventions to reduce perineal trauma have been studied (2). Pain, bleeding, and the need for wound healing are directly associated with the extent to which the genital tract trauma has occurred during delivery (3). Postpartum bleeding poses a threat to the health of the mother due to the large episiotomy incision, the extension of the tear, and delay in the episiotomy repair. Moreover, the damages to the perineum and the resulting pain can cause postpartum problems such as difficulty walking, sitting, nursing, and care for the newborn (4). Perineal damage not only causes physical damage but also results in the mother's emotional and psychological injury, and delayed healing of the wound due to poor anatomical outcomes, poor healing of the incision site, and increased perineal pain (5). As noted, perineal trauma following vaginal delivery can be associated with short- and long-term complications. The potential complications associated with vaginal birth are worrisome so any procedure that reduces the likelihood Trauma to the genital tract is suggested. Some have recommended routine perineal massage to reduce the incidence of perineal trauma during vaginal delivery. Perineal massage may increase the flexibility of the perineal muscles, thereby reducing muscle resistance, causing the perineum to stretch during labor without rupture and no need for an episiotomy (6).

Besides that, researchers are searching for ways that can reduce the severity of spontaneous perineal tears, some of which are concerned with the antenatal period, including the perineal message (7), and some others have used various techniques and maneuvers during labor (8). 

The perineal massage (PM) technique using a lubricant is a potential physiotherapeutic method applied for the active phase of labor, which can lead to muscle dilation and trauma prevention upon vasodilatation and increased blood flow to the area (9). Since the studies conducted on the effect of PM on the prevention of perineal damages, their consequences, and the repair of episiotomy are few, we decided to examine the effect of PM on the need for an episiotomy.

## Materials and methods

This study is a randomized, double-blind clinical trial that included nulliparous pregnant women aged 18 to 35 years referring to Um-al-Banin Educational-Therapeutic Hospital in Mashhad from July to October 2018. The study protocol was approved by the Committee of Ethics in Medicine affiliated with Mashhad University of Medical Sciences on 12 July 2017 (letter No.: IR.MUMS.fm.REC.1396.186).

Inclusion criteria comprised of singleton pregnant patients with a live fetus, occiput anterior presentation, 37-42 week of gestation, tendency for vaginal delivery, estimated baby weight below 4 kg, active stage of labor (first stage; 6 cm dilatation) at admission, and written informed consent for participation. Patients with posterior occiput, polyhydramnios, fetal distress, intrauterine death, prematurity, post-maturity, vacuum delivery, and reluctance to continue to participate in the study were excluded.

The number of samples in each group was calculated as 45. However, a total of 50 was considered given the calculated attrition of 10 percent. Eventually, one of the mothers in the control group was excluded from the study due to vacuum delivery, leaving 49 patients in the control group and 50 in the intervention group.

**Figure 1:**
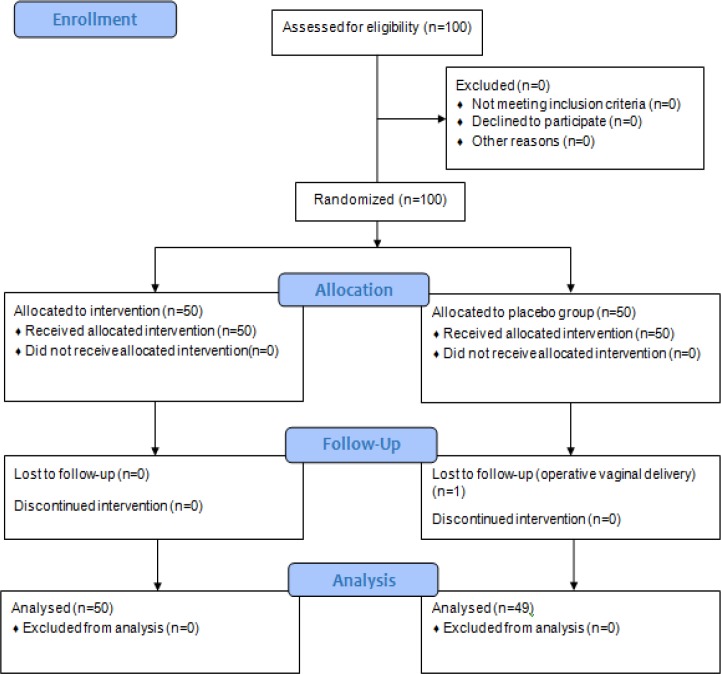
CONSORT 2010 Flow Diagram

A convenience sampling method was adopted. First, the researcher explained thoroughly the goals of the study to the women who referred to the hospital for vaginal delivery. After they signed written consent forms, the demographic characteristics data including age, education, weight, and gestational week were collected. The participants were randomly assigned to an intervention group and a control group based on a random allocation list generated by a software application. Subsequently, the intervention group or control group titles were written inside sealed envelopes and coded. They were pulled out from the envelopes sequentially according to the random allocation list during delivery whereby the patient was placed in either the intervention or control groups.

All the massages were given by one of the research colleagues (i.e., the fourth-year resident of gynecology) so that the manner and severity of the massage would be similar for all the mothers. This study is double-blind, and only the midwife doing the delivery was not aware of the group allocations.

The PM was performed for all the women in the intervention group at the active stage of labor (from 6 cm dilatation of the cervix to the completion of cervical dilatation) and the beginning of the second stage (after completion of cervical dilatation until the baby was extubated). Before the massage, the emptiness of the intestines and the bladder were assured. All the participants lied in the lithotomy position, and the massage began after the contraction severity of the muscles was reduced in the resting position. 

In the first phase of PM, the researcher wore gloves and rubbed two drops of lubricant on the fingers. She inserted fingers into the vagina 2-3 cm and pressed both sides of the vaginal wall, continuing for two minutes. The women had a minimum of 30 minutes of rest before the next massage session initiated. Four massage sessions were performed during the first stage of labor. At the start of the second stage of labor, the participants received a 10-minute massage. Control group women received the routine care of the hospital and underwent a routine vaginal examination.

After delivery, the two groups were examined for perineal damages. The frequency of episiotomy in the groups and its degree, the degree of spontaneous perineal tear and its degree, duration of the first and second stages of labor, and newborn’s weight and head circumference were measured and recorded by the resident researcher in a checklist.

In the end, the data were analyzed in the SPSS software (version 16) and presented in tables and charts using descriptive statistical indices in terms of frequency. In case the data were distributed normally, independent t-test was used to compare the quantitative variables between the two groups; otherwise, the Mann-Whitney test was used. Chi-Square test was applied to compare qualitative variables between the two groups as well as Fisher's exact test, if necessary. In all calculations, a significant level of 0.05 was considered.

## Results

In this study, 99 patients were included of whom 49 were in the control group and 50 in the intervention group. As shown in tables 1, 2, and 3, there were no significant differences between the two groups in terms of demographic and confounding variables.

**Table 1:** The frequency of demographic and confounding variables

	**Groups**	**Positive** **Frequency (percent)**	**Negative** **Frequency (percent)**	**P-** **value**
Hypertension	Control	47 (95.9)	2 (4.1)	0.02*
	Perineal message	50 (100.0)	0 (0.0)	
	Total	97 (98.0)	2 (2.0)	
Diabetes	Control	45 (91.8)	4 (8.2)	0.04*
	Perineal message	48 (96.0)	2 (4.0)	
	Total	93 (93.9)	6 (6.1)	
Hypothyroid	Control	47 (95.9)	2 (4.1)	1.0*
	Perineal message	48 (96.0)	2 (4.0)	
	Total	95 (96.0)	4 (4.0)	
Education level		High school diploma or below	Tertiary	0.02*
	Control	45 (91.8)	4 (8.2)	
	Perineal message	49 (98.0)	1 (2.0)	
	Total	94 (94.9)	5 (5.1)	
Gender of the infant	Girl Frequency (percent)	Boy Frequency (percent)		0.02*
	Control	25 (51.0)	24 (49.0)	
	Perineal message	20 (40.0)	30 (60.0)	
	Total	45 (45.5)	54 (54.5)	

* Fisher’s exact test and Chi-square test

**Table 2:** Medians of demographic and confounding variables

**Variables under study**	**Groups**	**Number**	**Median**	**Minimum**	**Maximum**	**P-value**
Gestational age (week)	Control	49	40.0	38.0	42.0	0.5*
	Perineal message	50	40.0	37.0	42.0	
Head circumference	Control	49	34.0	30.0	37.0	0.4*
	Perineal message	50	34.0	30.0	36.5	

* Mann-Whitney test

Other results showed that the need for episiotomy was significantly different between the two groups (p = 0.05) and that the need for episiotomy was lower in the group that received the PM (Table 4). Moreover, the rate of spontaneous perineal tears differed significantly between the two groups (p = 0.05) where the tear’s frequency was more substantial in the PM group (Table 4). However, the degree of spontaneous perineal tear in the 20 mothers who did not need episiotomy was not significantly different between the two groups (p = 0.5) (Table 4). Also, the degree of perineal tear in mothers who needed an episiotomy (n = 79) did not reach statistical significance in the groups (p = 0. 1) (Table 4). Moreover, the first-degree tears were more prevalent than the second-degree tears in mothers of the PM group who did not require episiotomy (n = 14 mothers) and in mothers who had a spontaneous tear.

The median duration of the active phase of labor until the completion of this phase in the PM group was lower than in the control group. However, according to the results of the statistical test, this difference was not statistically significant (p = 0.3) (Table 5). The mean duration of the second stage of labor in the control and PM groups were 55 and 45 minutes, respectively. The results showed that there was a significant difference between the groups in terms of the median scores of the second stage of labor (p = 0.002) where the mean time of completing the active phase of labor before delivery was reduced in the PM group (Table 5). Finally, it was found that there was no significant difference between the Apgar mean scores at the first minute (p = 0.1) and the fifth minute (p = 0.3).

## Discussion

Based on the results of the present study, the need for episiotomy in the group receiving PM was significantly lower than that of the control group, attributably because of the proper massage, which leads to increased blood flow, elasticity, and perineal tissue softness. Shahoei et al. studied the effect of PM during the second stage of labor on the frequency of perineal tears, episiotomy, and perineal pain in nulliparous women, indicating that the antenatal massage could reduce the need for episiotomy, perineal damages, and perineal pain. The PM practiced in the study of Shahoei et al. and ours were similarly in U form; however, the massage in Shahoei et al.’s study was given only in the second stage of labor (10). The results of Demirel and Golbasi’s study (2015) on the effect of PM on episiotomy and perineal tear showed that 31% of the intervention group members and 69.7% of the control group members needed an episiotomy, suggesting that PM reduces the rate of episiotomy (11). Given the fact that the PM also reduced the need for episiotomy in our study, our results correspond with those of Shahoei et al. (2017) and Demirel and Golbasi’s (2015) studies.

In similar lines with the results of the present study, Beckmann and Garrett (2006) investigated the effect of PM on the reduction of perilial tear rate in Australia, showing that the frequency of episiotomy was lower in the PM group.

**Table 3:** Mean scores of demographic and confounding variables

**Variables under study**	**Groups**	**Number**	**Mean**	**Standard deviation**	**P-value**
Mothers’ age	Control	49	23.87	4.86	0.1*
	Perineal message	50	22.46	3.94	
Mothers’ weight	Control	49	68.22	11.33	0.7*
	Perineal message	50	68.80	11.00	
Newborns’ weight	Control	49	3.16	428.15	0.2*
	Perineal message	50	3.25	362.93	

* T-test

**Table 4:** Primary outcomes in mothers with and without antenatal massage in the active phase of labor

**Primary outcome**		**Yes** **Frequency ** **(percent)**	**No** **Frequency ** **(percent)**	**P-** **value**
Degree of need for episiotomy	Control	6 (12.2)	43 (87.8)	0.05*
	Perineal message	14 (28.0)	36 (72.0)	
	Total	20 (20.2)	79 (79.8)	
Spontaneous perineal tear	Control	43 (87.8)	6 (12.2)	0.05*
	Perineal message	36 (72.0)	14 (28.0)	
	Total	79 (79.8)	20 (20.2)	
Degree of the perineal tears in mothers without the need for episiotomy	Control	6 (100.0)	0 (0.0)	0.5*
	Perineal message	11 (78.6)	3 (15.0)	
	Total	17 (85.0)	3 (15.0)	
Degree of the perineal tears in mothers with the need for episiotomy	Control	4 (9.3)	39 (90.7)	0.1*
	Perineal message	8 (22.2)	28 (77.8)	
	Total	12 (15.2)	67 (84.8)	

* Fisher’s exact test and Chi-square test

They also found that the decrease was more significant in nulliparous than multiparous women, where the pain relief was also reported for up to three months after delivery. They concluded that antenatal massage during pregnancy reduced the risk of perineal damage, episiotomy, and the subsequent postpartum perineal pain (12). Besides, Labrecque et al. (1999), in a study on the effect of PM during pregnancy on postpartum perineal symptoms, found that 24% of women in the PM group did not suffer any perineal damage. Therefore, it was concluded that antenatal massage is useful for women who are experiencing their first vaginal delivery (13).

Other results of the present study showed that the tear rate was significantly higher in the group receiving the PM than the control group. There was no significant difference in terms of the degree of the perineal tear between the mothers who did not need episiotomies and those who needed an episiotomy. However, the second-degree perineal tear was of a lower frequency among mothers who received PM. Therefore, it seems that PM does not have an effect on the number of tears but reduces their depth. Shipman et al. suggest that antenatal PM during the third trimester of pregnancy is associated with benefits such as reduced need for episiotomy, second- and third-degree tears, and instrumental delivery where the impacts are higher for women above 30 years of age (14). Their study results in terms of the effect of PM on the reduction of perineal tears during delivery are consistent with the results of our study. Nonetheless, the two studies differ as PM in our study was practiced only during the first and second stages of labor, while in Shipman et al.’s study, it was performed only during the third trimester of pregnancy, which may justify the non-significance of the statistical test’s result.

In this regard, Vendittelli et al. argue that antepartum PM is effective in preventing severe perineal damages but that further studies and patient satisfaction are needed to prove this (15). In a systematic review, Eason et al. reported that PM prevents perineal trauma in the last weeks of pregnancy (16).

**Table 5:** Secondary outcomes in mothers with and without perineal massage in the active phase of labor per minute

**Variables **	**Group**	**Number**	**Median**	**Minimum**	**Maximum**	**P-value**
First stage of labor	Control	49	270	155	390	0.3
	Perineal message	50	240	165	480	
Second stage of labor	Control	49	55.0	30.0	120.0	0.002*
	Perineal message	50	45.0	20.0	120.0	

* Mann-Whitney test

In a clinical trial by Albers et al. (2005) on the procedures to reduce genital tract trauma during spontaneous vaginal birth, the participants were divided into three groups: perineal warm compress, midwife massaging with lubricant, and no-intervention until the head was crowning. The prevalence of perineal and vaginal damage was not significantly different in the groups. The authors stated that the central predictor of perineal damage was antenatal and macrosomia of the fetus and that the mother's sitting position during childbirth plays a preventive role against perineal damage (8). It is likely that the reason for the difference between the results of our study and Albers et al.’s study lies with the difference in patient position and the duration of the first and second stages of labor, which reduced the opportunity for sufficient massage and led to poorer control of the perineum.

In another study, Mei-dan et al. (2008) found no significant difference between the intervention and control groups regarding the effect of PM on perineum health (17), which can be attributed to racial differences or differences in the quality and duration of the massage.

However, Labrecque et al. reported the mean and standard deviation of the duration of the second stage of labor in the massage and control groups as 89.9 ± 36.4 and 85.9 ± 60.7, respectively (13). Also, Boland Hemmat et al. reported 56.8 ± 26.9 and 52.7 ± 26.5 minutes for the duration of the second stage of labor in the massage and control groups, respectively (18), whose findings are not consistent with our results. This suggests that PM does not reduce the duration of the second stage of labor in women, the inconsistency being attributable to the difference in the head-pelvis alignment.

Generally, the preparation for pregnancy begins with the secretion of the progesterone and relaxin hormones during pregnancy; these hormones help muscles and joints to soften and stretch; this process takes place without exception in the entire body, pelvic floor, and perineum. It seems that a slow and soft massage can increase the perineal stretchability upon increased blood circulation (13).

## Conclusion

The results of this study indicated the effect of PM on the reduction of the need for an episiotomy and its ineffectiveness on the rate of tear. However, the results also showed that among the PM mothers who did not require episiotomy and experienced a spontaneous tear, first-degree tear of the perineum was of a higher frequency than the second-degree tear. The median duration of the second stage of labor was also significantly reduced in the PM group. Overall, considering the results of this study and their comparison with the results of other studies, we may suggest PM in the active phase and the second phase of labor as a safe, simple, and low-cost measure, which can reduce perineal damages resulting from delivery. It is also recommended to study the effect of PM in the third trimester of pregnancy on the frequency of episiotomy and perineal tear during labor in nulliparous women in future studies.
